# Human Mast Cells (HMC-1 5C6) Enhance Interleukin-6 Production by Quiescent and Lipopolysaccharide-Stimulated Human Coronary Artery Endothelial Cells

**DOI:** 10.1155/2012/274347

**Published:** 2012-01-26

**Authors:** Damandeep S. Walia, Mukut Sharma, Vineesh V. Raveendran, Jianping Zhou, Ram Sharma, Daniel J. Stechschulte, Kottarappat N. Dileepan

**Affiliations:** ^1^Division of Allergy, Clinical Immunology, and Rheumatology, Department of Internal Medicine, University of Kansas Medical Center, Kansas City, KS 66160, USA; ^2^Kansas City, Veterans Affairs Medical Center, Kansas City, MO 64128, USA

## Abstract

We examined the effect of intact human mast cells (HMC-1 5C6) and their selected mediators on interleukin-6 (IL-6) production and bone morphogenetic protein-2 (BMP-2) expression in human coronary artery endothelial cells (HCAEC) in the presence and absence of lipopolysaccharide (LPS). Scanning electron microscopy showed that HMC-1 5C6 cells adhere to HCAEC in cocultures. Addition of HMC-1 5C6 cells markedly enhanced the IL-6 production by quiescent and LPS-activated HCAEC even at the maximal concentration of LPS. Furthermore, mast cell-derived histamine and proteases accounted for the direct and synergistic effect of mast cells on IL-6 production that was completely blocked by the combination of histamine receptor-1 antagonist and protease inhibitors. Another novel finding is that histamine was able to induce BMP-2 expression in HCAEC. Collectively, our results suggest that endotoxin and mast cell products synergistically amplify vascular inflammation and that histamine participates in the early events of vascular calcification.

## 1. Introduction

Mast cells are normal constituents of the vessel wall and are primarily located in the connective tissue matrices. Increasing evidence suggests an important role for mast cells in vascular inflammation and atherosclerosis [1–4], fibrosis [5], osteoporosis [6], and host defense [7]. The adventitia of coronary arteries of patients with atherosclerotic plaques contains increased number of mast cells [1–3, 8–10]. An increase in the number of tissue mast cells is also found to be associated with thrombus formation [3]. Mast cell granules have been identified within endothelial cells *in vivo* [11] and are known to cause proliferation of human microvascular endothelial cells [12]. Furthermore, mast cell granule remnants can bind to low-density lipoproteins (LDL) and enhance their uptake by macrophages leading to the development of foam cells [13, 14]. We have previously shown that granules isolated from rat peritoneal mast cells interact with human endothelial cells in culture and enhance lipopolysaccharide- (LPS-) induced production of interleukin-6 (IL-6) and IL-8 [15–17]. These findings suggest that mast cells play an important role in endothelial cell activation and vascular inflammation. However, the interaction of human mast cells with human endothelial cells and its impact on inflammatory responses has not been evaluated.

 Vascular endothelium regulates many aspects of the inflammatory response in addition to its well-recognized function as a protective barrier between blood and tissues. Activation of endothelial cells and resultant enhancement in the production of proinflammatory cytokines, prostanoids, and the expression of adhesion molecules are critical factors for the immune response and host defense as well as for the progression of inflammatory diseases. The cascade of endothelial activation can be initiated by both endogenous agonists such as inflammatory cytokines, lipid mediators, histamine, and proteases, and by exogenous agents including microbial agents. We have shown that the cell wall components of both gram-positive and gram-negative bacteria can induce inflammatory responses in human endothelial cells [15–18] suggesting an important role for infectious agents in initiating vascular inflammation.

Mast cells synthesize and secrete many vasoactive substances and inflammatory mediators including histamine, proteases (tryptase, chymase, and carboxypeptidase A) prostaglandin D2, leukotrienes, heparin, and cytokines [19–23]. Histamine and tryptase are two major secretory products of mast cells. The involvement of histamine in vascular disease is suggested by its elevated levels in the coronary circulation of patients with variant angina [24] and an increased expression of histamine H1receptor (H1R) in atherosclerotic lesions [25]. Previous reports from our laboratory show that histamine induces the expression of cyclooxygenase-2 and IL-6 mRNA and the production of PGI_2_, PGE_2_, and IL-6 [18, 26, 27]. A number of reports have also documented the role of mast cells in the formation of foam cells [13, 14] and calcification of plaques [9, 10]. Bone morphogenetic protein-2 (BMP-2**) **is a key molecule in the calcification cascade and its expression in endothelial cells is induced by TNF-*α* or by mechanical stimuli [28, 29]. Although mast cells are involved in atherosclerotic plaque development [9, 10], direct effect of mast cell components on the expression of BMP-2 has not been explored. Therefore, the objective of the present study was to evaluate the effect of intact human mast cells and their products on the regulation of inflammatory and calcification responses in naïve and LPS-activated HCAEC.

## 2. Materials and Methods

### 2.1. Cells

Human coronary artery endothelial cells and endothelial cell growth medium (EGM-2MV) were obtained from Cambrex (San Diego, CA). Human mast cell line, subclone HMC-1 5C6 (derived from HMC-1 cells) was kindly provided by Dr. Michel Arock (Ecole Normale Supérieure de Cachan, Cachan, France).

### 2.2. Culture of Endothelial Cells

HCAEC were grown in EGM-2MV containing 1 *μ*g/mL hydrocortisone acetate, 50 ng/mL gentamycin, 50 *μ*g/mL amphotericin B, and the recommended concentrations of human epidermal growth factor, vascular endothelial growth factor, human fibroblast growth factor, recombinant insulin-like growth factor-1, ascorbic acid, and 5% fetal bovine serum as described previously [15, 16]. At confluence, cells were detached from the culture flasks using trypsin-EDTA, washed twice, and resuspended in fresh EGM-2MV. The cells used in all experiments were between passages three and six.

### 2.3. Mast Cells

HMC-1 5C6 cells were grown in Iscove's Modified Dulbecco's medium containing 10% FBS and 50 *μ*M of 2-mercaptoethanol at 37°C and 5% CO_2_. When applicable, mast cells were pretreated overnight with protease inhibitor cocktail (Sigma Chemical Co., St. Louis, Mo) containing 4-(2-aminoethyl)benzenesulfonyl fluoride hydrochloride 1 mM, bestatin 40 *μ*M, E-64 14 *μ*M, leupeptin 20 *μ*M, pepstatin 15 *μ*M, and aprotinin 0.8 *μ*M. Protease inhibitor-treated mast cells were incubated with HCAEC and washed twice with EGM-2MV. Mast cells treated with EGM-2MV containing 1% DMSO were used as controls since DMSO was used as the vehicle to resuspend the protease inhibitor cocktail.

### 2.4. Determination of IL-6 Production

HCAEC were seeded onto 96-well cell culture plate (1 × 10^4 ^cells/well) and allowed to adhere for 16 to 18 h. Following adherence, endothelial cell monolayers were incubated with mast cells or lysates derived from equivalent counts of mast cells in the presence or absence of LPS in a final volume of 0.2 mL at 37°C under 5% humidified CO_2_. After the indicated incubation period, culture supernatants were harvested, appropriately diluted, and assayed for IL-6 levels by ELISA [16–18].

### 2.5. Real-Time PCR (qRT-PCR) Analysis of the Expression of BMP-2

HCAEC monolayers were incubated with medium, histamine, cetirizine, histamine + cetirizine, or with LPS (10 and 100 ng/mL) for 2 h. Following incubation, supernatant was removed and total RNA was isolated using RNeasy Mini Kit (Qiagen, Valencia, CA), according to the manufacturer's protocol. Total RNA (1 *μ*g) was reverse transcribed into first-strand cDNA using High-Capacity cDNA Achieve Kit (Applied Biosystems, Foster City, CA) following the manufacturer's procedure. The primers used for SYBR Green real-time RT-PCR were designed using the Primer Express Software v3.0 (Applied Biosystems, Foster City, CA). The sequences of primers used for real-time PCR analyses of various genes are, *human BMP-2:* forward- TGCTAGTAACTTTTGGCCATGATG; reverse- GCGTTTCCGCTGTTT GTGTT, *human *β* Actin:* forward- CCAGCTCACCATGGATGATG; reverse- ATGCCGGAGCCGTTGTC.

### 2.6. Western Blot Analysis of BMP-2

HCAEC monolayers were incubated with medium, histamine, LPS, or histamine + LPS for 2 or 24 h. After the incubation, the supernatant was removed and the cells were sonicated in the lysis buffer containing 1% Triton X100, protease, and phosphatase inhibitors, and they were centrifuged. An aliquot of the lysate was transferred to sample wells in 10% Mini-PROTEAN precast gels (BioRad, Hercules, CA, USA). SDS-PAGE was carried out in Tris-glycine buffer at 60 volts for 120 min. Separated proteins were transferred to PVDF membranes by electroblotting at 90 volts for 60 min. Fat-free milk powder (5%) was used as the blocking agent. Rabbit-anti-human BMP-2 polyclonal antibody (1 : 500 dilution) was used as the primary antibody (ab-14933, Abcam, Cambridge, MA, USA) and HRP-conjugated goat-anti-rabbit antibody (1 : 3,000 dilution) as the secondary antibody (ab-6721, Abcam). *β*-Actin, the loading control, was detected using anti-*β*-actin AC-15 (A5441, Sigma, 1 : 10,000 dilution) and HRP-conjugated goat-anti-mouse IgG antibody (170–5047, BioRad, 1 : 5,000 dilution) as the primary and secondary antibodies, respectively.

### 2.7. Electron Microscopic Analysis of Mast Cell-Endothelial Cell Interaction

Confluent HCAEC were cultured for 24 h in the presence or absence of HMC-1 5C6 cells at a mast cell-endothelial cell ratio of 1 : 2. After 24 h, cells were washed twice with fresh phosphate buffered saline. Washed cells were fixed in 2% glutaraldehyde and processed for scanning electron microscopy. For transmission electron microscopy, HMC-1 5C6 cells were sedimented by centrifugation. The pellet was washed and fixed in 2% glutaraldehyde and processed at the University of Kansas Medical Center Electron Microscopy Core Facility.

### 2.8. Statistical Analysis

Student's unpaired *t*-test or two-way ANOVA were adopted for statistical analyses as applicable. Results are represented as mean ± SEM and *P* < 0.05 is considered significant.

## 3. Results

In order to examine the effect of human mast cells, we compared the direct and synergistic effects of HMC-1 5C6 on IL-6 production by HCAEC.

### 3.1. Effect of HMC-1 5C6 on IL-6 Production by HCAEC Stimulated with LPS

**Figure 1:**
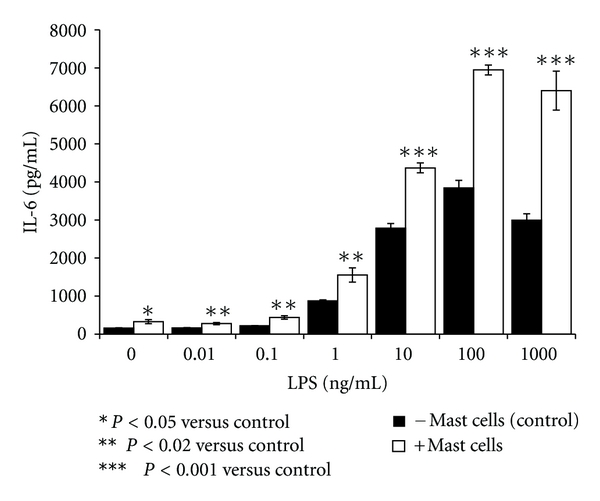
Mast cells potentiate LPS- induced IL-6 production by HCAEC. Endothelial cells (10,000 cells/well) were grown for 24 h in the presence or absence of HMC-1 5C6 (5000 cells/well) and stimulated with indicated concentrations of LPS. Following incubation, the cell culture supernatants were analyzed for IL-6 by ELISA. Results presented are mean ± SEM of quadruplicate determinations of a representative experiment. Similar results were observed in two additional experiments.

The effects of HMC-1 5C6 on IL-6 production by unactivated or LPS-treated HCAEC are presented in Figure 1. Unactivated HCAEC produced relatively low levels of IL-6 in a 24 h culture. However, LPS stimulated the production of IL-6 by HCAEC in a dose-dependent manner which attained a plateau at 100 ng/mL LPS. Presence of HMC-1 5C6 in the coculture, at an endothelial cell: mast cell ratio of 1 : 1 resulted in a marked potentiation of LPS-induced IL-6 production at all concentrations of LPS tested. It is of interest that mast cells alone induced a small but significant increase in IL-6 production by HCAEC. The amplification of LPS-induced cytokine production by HMC-1 5C6 was evident at LPS concentrations as low as 0.1 ng/mL and at higher doses (100 and 1000 ng/mL LPS). Once again, increased levels of IL-6 cannot be attributed to the IL-6 production by HMC-1 5C6 because these cells did not generate detectable amounts of IL-6 in the presence or absence of LPS (data not shown).

### 3.2. Effect of Endothelial Cell: Mast Cell Ratios on IL-6 Production

**Figure 2:**
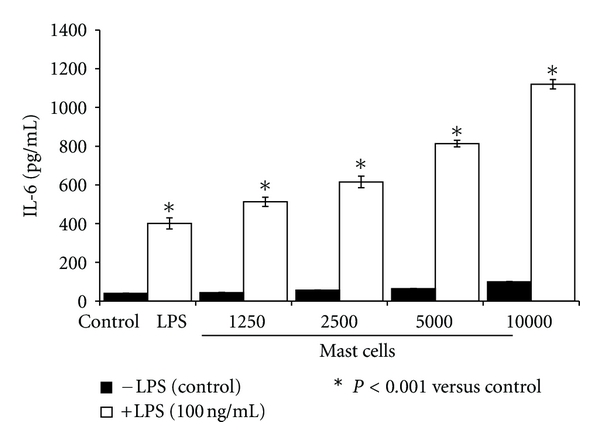
Mast cells dose-dependently potentiate LPS-induced IL-6 production by HCAEC. Endothelial cells (10,000 cells/well) were grown for 24 h in the presence of increasing number of HMC-1 5C6 cells and were activated with LPS (100 ng/mL) for a period of 24 h. After the incubation, culture supernatants were analyzed for IL-6 by ELISA. Results are mean ± SEM of quadruplicate determinations of a representative experiment. Similar results were observed in two other experiments.

As shown in Figure 2, addition of increasing counts of HMC-1 5C6 cells to a constant number of HCAEC resulted in progressive increase in the production of IL-6 both by unactivated and LPS-stimulated cells. The potentiating effect of HMC-1 5C6 cells on LPS-induced IL-6 production by HCAEC was linear with the number of mast cells present in the culture. The mast cell-mediated amplification of LPS-induced IL-6 production was approximately 300% of the control at the HCAEC: mast cell ratio 1 : 1.

### 3.3. Transmission and Scanning Electron Microscopy Studies

**Figure 3:**
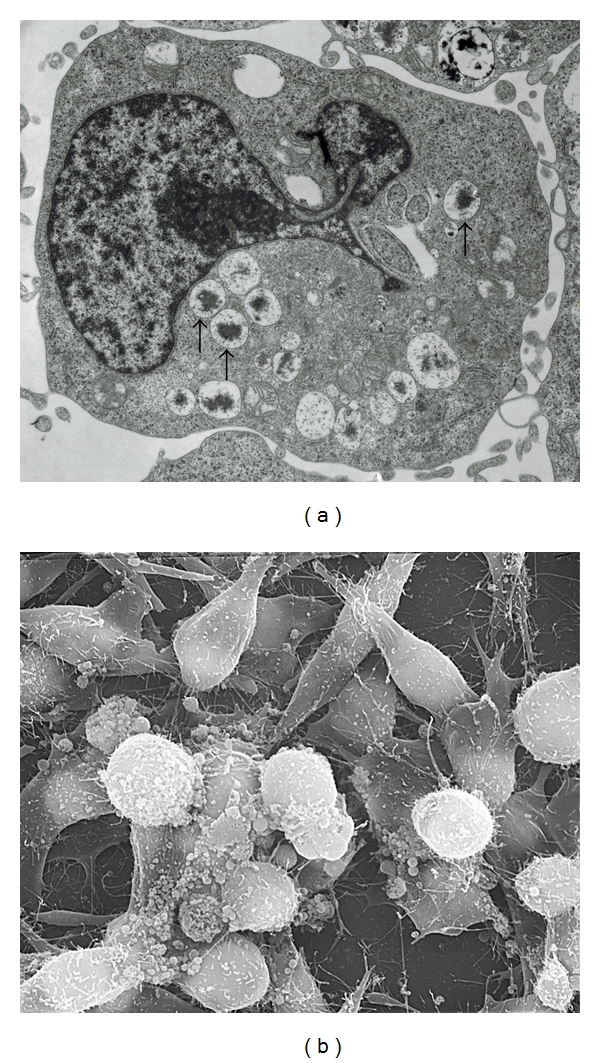
Transmission and scanning electron microscopy. The ultrastructural assessment of HMC-1 5C6 cells demonstrates the presence of cytopslasmic granules; (a) shows HMC-1 5C6 clone exhibited numerous electron-dense granules (arrows) indicating maturity of the cells (magnification 14,000X); (b) shows a scanning electron micrograph of HMC-1 5C6 in a typical co-culture experiment demonstrating endothelial cell-mast cell interaction (magnification 2600X).

The ultrastructural assessment of HMC-1 5C6 cells demonstrates the presence of cytoplasmic granules (Figure 3). The subclone HMC-1 5C6 (Figure 3(a)) exhibited several electron-dense granules (arrows), indicating maturity of granules in these cells. The scanning electron microscopy following co-culture of HCAEC and HMC-1 5C6 (Figure 3(b)) demonstrates the interaction between these two cell types in a 24 h coculture.

### 3.4. Effect of Intact Mast Cells and Cell Lysate

Results presented in Table 1 demonstrate that coincubation of HCAEC with HMC-1 5C6 cells resulted in endothelial cell activation. This finding was consistent with our previous results using rat mast cell granules (MCG) [16, 17]. As expected, incubation with LPS caused significant increase in IL-6 production by endothelial cells. The 5C6 subclone-induced cytokine production was markedly enhanced (300%) by the presence of LPS (100 ng/mL) indicating synergy between human mast cells and LPS on endothelial cell activation. In addition, lysates of HMC-1 5C6 subclone showed synergistic effects with LPS on endothelial cell IL-6 production. The enhanced levels of IL-6 were not derived from the mast cell lines because incubation of HMC-1 5C6 subclone with or without LPS did not generate IL-6 (data not shown). Furthermore, the mast cell lysate did not contain detectable amounts of IL-6 (Table 1).

### 3.5. Role of Histamine and Proteases on the Synergy between Mast Cells and LPS

**Figure 4:**
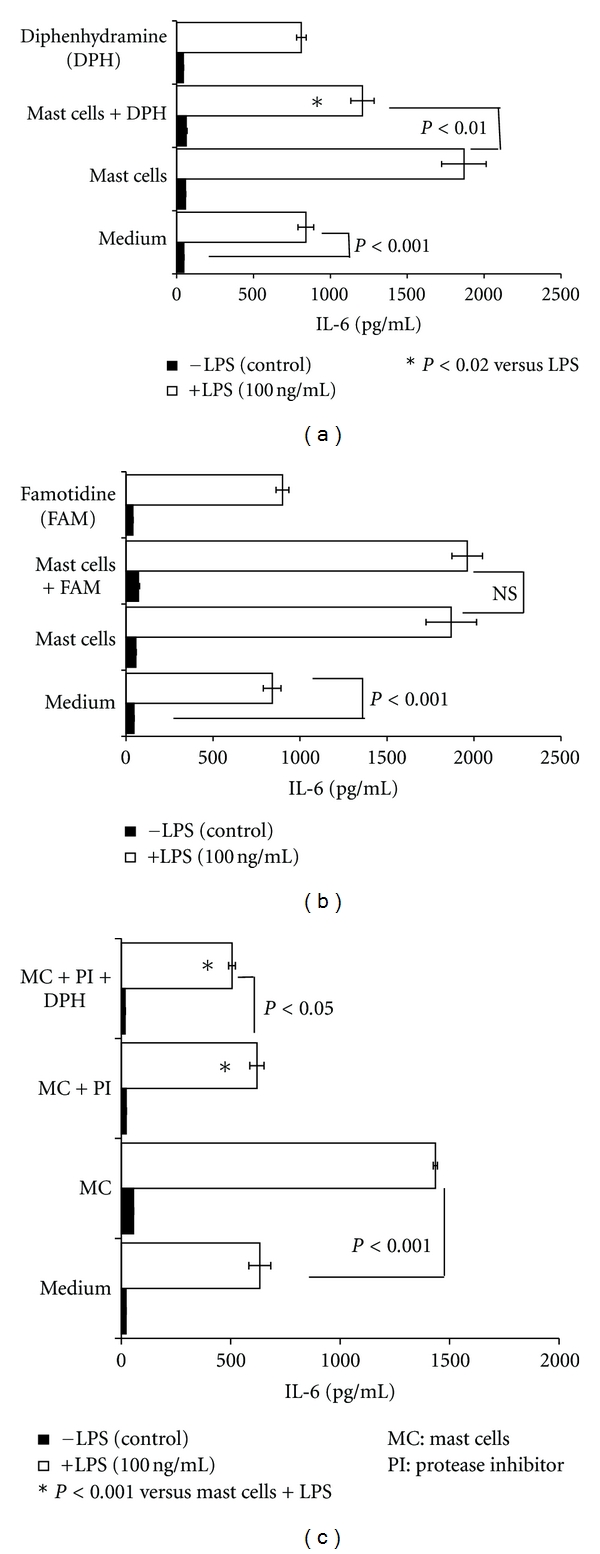
Combination of H1R antagonist and protease inhibitors (PI) abrogates mast cell- (MC-) mediated potentiation of IL-6 production by LPS-stimulated endothelial cells. Endothelial cells (10,000 cells/well) were grown for 24 h in the presence of mast cells (5000 cells/well) and were activated with LPS (100 ng/mL) for a period of 24 h. To determine the effect of H1R or H2R antagonists, endothelial cells were incubated with 10 *μ*M diphenhydramine (DPH) (a) or 10 *μ*M famotidine (FAM) (b) for 30 min before addition of HMC-1 5C6. To determine the contribution of mast cells proteases (c), HMC-1 5C6 were pretreated with protease inhibitor (PI) cocktail overnight, washed twice, and then added to endothelial cell monolayers. After 24 h incubation, the culture supernatants were analyzed for IL-6 by ELISA. Results are mean ± SEM of quadruplicate determinations of a representative experiment. Similar results were observed in two other experiments.

**Figure 5:**
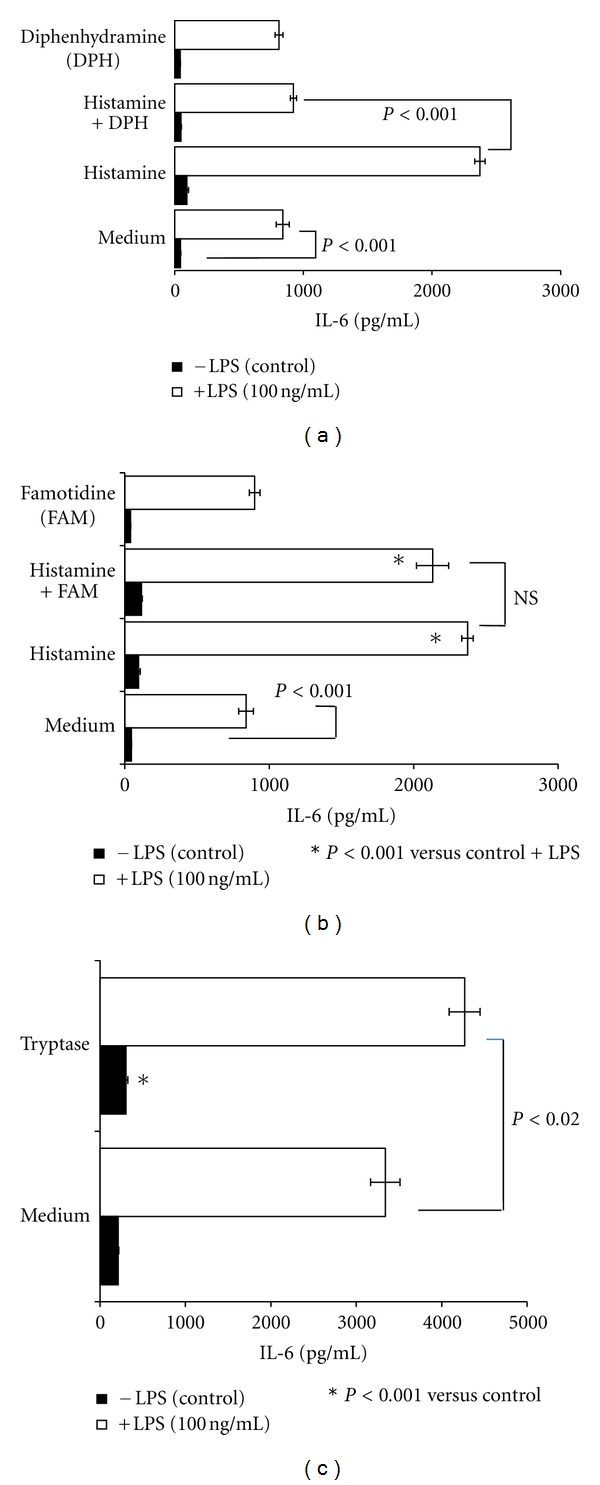
Histamine and Tryptase induce potentiation of IL-6 production by LPS-stimulated endothelial cells. Endothelial cells (10,000 cells/well) were incubated with histamine (10 *μ*M) (a and b) or human tryptase (50 mU/mL) (c) in the presence or absence of LPS (100 ng/ml) for 24 h. After the incubation, culture supernatants were analyzed for IL-6 by ELISA. To determine the effect of H1R or H2R antagonists, endothelial cells were incubated with 10 *μ*M diphenhydramine (DPH) (a) or 10 *μ*M famotidine (FAM) (b) for 30 min before addition of histamine. Results are mean ± SEM of quadruplicate determinations of a representative experiment. Similar results were observed in two other experiments.

Histamine is a major constituent of the mast cell. The effect of histamine on endothelial cells is primarily mediated through H1R [18, 26, 27, 30]. Therefore, to determine whether histamine derived from HMC-1 5C6 mediates the amplification of the effect of LPS on HCAEC, studies were conducted in the presence of 10 *μ*M diphenhydramine (H1 receptor antagonist) or 10 *μ*M famotidine (H2 receptor antagonist). As shown in Figure 4(a), 10 *μ*M diphenhydramine significantly inhibited the HMC-1 5C6-induced potentiation of LPS effect on IL-6 production. In contrast, H2 receptor antagonist, famotidine, did not inhibit the potentiating effect of mast cells on LPS response (Figure 4(b)). The fact that exogenous histamine (10 *μ*M) was able to synergize the effect of LPS on IL-6 production by HCAEC (Figure 5(a)) further confirms the role of mast cell-derived histamine (Figure 4(a)). Once again, the histamine-mediated amplification of LPS-activation was abrogated by the H1R antagonist and not by the H2R antagonist. Neither diphenhydramine nor famotidine alone had any effect on the LPS-induced IL-6 production by endothelial cells (Figures 4 and 5). The concentrations of histamine and the receptor antagonists used here were selected based on optimum stimulatory and inhibitory effects, respectively [18, 26, 27].

Serine proteases are present in the mast cells [20–23] and are known to activate endothelial cells [17, 31]. We determined the effect of mast cell-derived protease(s) on the LPS-stimulated production of IL-6 by HCAEC. First, mast cells (MC) were incubated with a cocktail of protease inhibitors (PI) for 18 h (MC + PI) and washed twice to remove PI. The mast cell suspension free of PI was incubated with HCAEC cultures.  Figure 4(c) shows that addition of untreated mast cells potentiated the effect of LPS as anticipated. In contrast, mast cells pretreated with protease inhibitors did not potentiate the LPS-stimulated production of IL-6. Interestingly, addition of 10 *μ*M diphenhydramine to pretreated mast cells resulted in a small but significant (*P* < 0.05) reduction in the effect of MC + PI (Figure 4(c)). In concordance with these findings we noted that addition of human tryptase (50 mU/mL) potentiated the LPS-induced IL-6 production by HCAEC (Figure 5(c)).

### 3.6. Effect of Histamine on BMP-2 Expression

**Figure 6:**
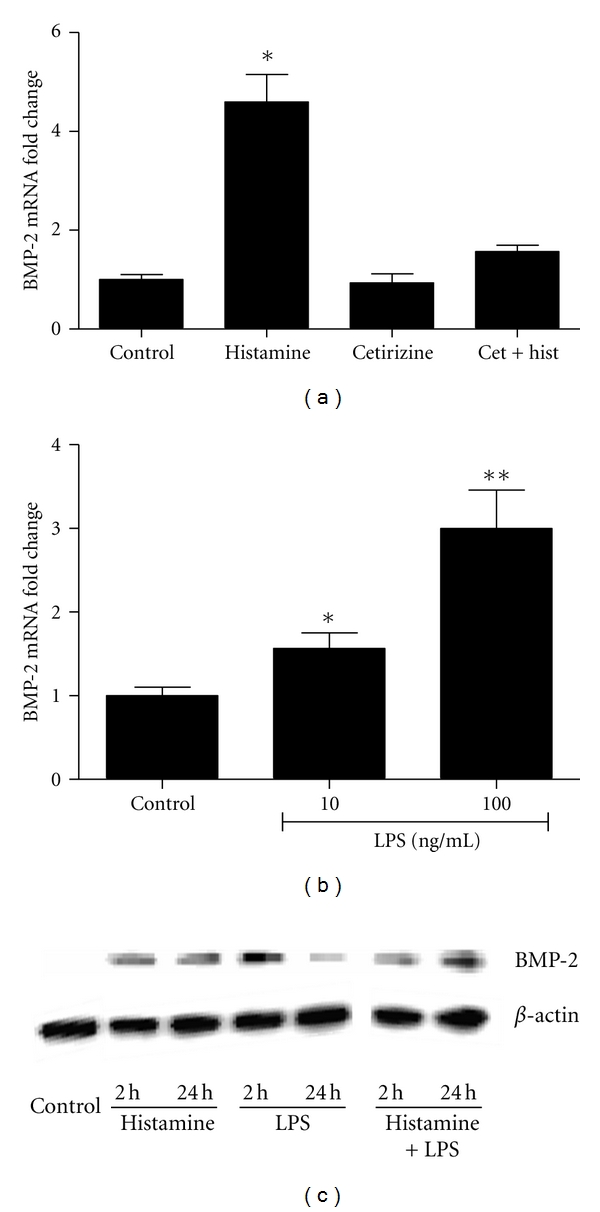
Histamine and LPS induce expression of BMP-2 mRNA. Endothelial cells were incubated with medium (control), histamine, histamine + cetirizine (a), or with 10 or 100 ng/mL LPS (b) for 2 h. Total RNA was extracted, reverse transcribed and, analyzed using specific primer sets for BMP-2 using real-time RT-PCR with *β*-actin as the housekeeping gene (a and b). Results were analyzed and normalized using *β*-actin and expressed as fold changes over the control. (c) Endothelial cells were incubated with medium, histamine, LPS, or histamine + LPS for 2 or 24 h, and Western blotting was carried out to determine the expression of BMP-2 protein as described in details in the Section 2.

Vascular calcification is one of the complications of chronic inflammatory diseases. BMP-2 is a powerful osteogenic morphogen which belongs to TGF-*β* superfamily and plays a central role in bone remodeling and calcification of atherosclerotic plaques [32–36]. In order to determine whether histamine has a role in the regulation of BMP-2, HCAEC monolayers were treated with histamine (10 *μ*M) or LPS (100 ng/mL) and the expression of BMP-2 mRNA and protein were determined using qRT-PCR and Western blot analysis, respectively. As shown in Figure 6(a), incubation of HCAEC with histamine resulted in 4-to 5-fold increase in BMP-2 mRNA expression which was completely abrogated by cetirizine, an H1R antagonist. Incubation of HCAEC with LPS also led to increased expression of BMP-2 mRNA in a concentration-dependent fashion (Figure 6(b)). Western blot analysis of the total protein in cell lysates showed upregulation of BMP-2 expression by histamine within 2 h which sustained up to 24 h. In contrast, LPS-induced expression of BMP-2 protein was transient. Interestingly, the presence of histamine appears to block the decrease in BMP-2 protein noted with LPS alone at 24 h time point.

## 4. Discussion

 The results obtained in this study demonstrate for the first time that coincubation of HCAEC and human mast cell subclone HMC-1 5C6 leads to enhanced production of IL-6 by both quiescent and LPS-stimulated HCAEC. The enhanced levels of IL-6 were not derived from mast cells because incubation of HMC-1 5C6 without HCAEC did not produce IL-6 in the presence or absence of LPS (data not shown). Furthermore, IL-6 was not detected in HMC-1 5C6 lysates confirming that these cells did not contribute to the observed increase in IL-6. Additional results revealed that HMC-1 5C6-derived histamine and serine proteases mediate IL-6 production directly as well as synergistically with LPS. The present study also demonstrates that histamine induces the expression of the osteogenic protein BMP-2 in HCAEC suggesting an important role for mast cells in vascular calcification.

Our laboratory has previously demonstrated synergistic interaction between bacterial products and rat mast cell granules on endothelial cells resulting in amplified production of IL-6 and IL-8 [15–17]. In agreement with this observation, human mast cell line HMC-1 5C6 at various concentrations corresponding to mast cell-to-endothelial cell ratios of 1 : 8, 1 : 4, 1 : 2, and 1 : 1 stimulated IL-6 production by HCAEC (Figure 2). In addition, mast cells potentiated the effect of LPS at all the concentrations tested confirming a synergy between the mast cell and LPS. The ability of this human mast cell line to act in concert with bacterial agent to modulate endothelial cell function has important clinical significance.

Scanning electron microscopy revealed that HCAEC and HMC-1 5C6 adhere to each other in the co-culture (Figure 3). Since mast cell lysates also showed a similar effect on HCAEC (Table 1), we submit that an adherence between intact mast cells and endothelial cells is not a prerequisite for the mast cell-mediated endothelial activation. Whether this cell-to-cell interaction involves selective adhesion molecules or receptors on the endothelial cells is unknown. Previous transmission electron micrograph studies have shown that mast cell-released granules (MCG) are internalized by cultured human umbilical vein endothelial cells (HUVEC) and remain morphologically intact for at least 24 h [15]. This phenomenon of mast cell transgranulation has been previously observed in human tissue [11]. The retention of structurally intact MCG after being internalized by endothelial cells is in contrast to the rapid degradation observed in macrophages [37]. The strategic location of mast cells in the vessel wall and their demonstrated ability to modulate endothelial cell functions emphasize the importance of an active communication between these two cell types. Transmission electron microscopy of the HMC-1 5C6 clone exhibited electron-dense particles in the granule compartments (Figure 3). These electron-dense particles were not detected in the parent HMC-1 cell line [38]. Thus, the HMC-1 5C6 clone appears to be functionally mature and suitable for evaluating mast cell functions.

Among the secreted products of the mast cell, histamine and proteases are the most extensively studied molecules. On a weight and molar basis, neutral proteases are the major exocytosed protein constituents of activated mast cells [20]. Tryptase, chymase, and carboxypeptidase A, represent the three major classes of proteases stored in the granules of mast cells [20–23]. Present results show that mast cell-induced activation of HCAEC was partly due to serine proteases. Pretreatment of mast cells with a cocktail of protease inhibitors (PI) partially abrogated the stimulation of IL-6 production by quiescent and LPS-activated HCAEC (Figure 4(c)) suggesting that proteases released by mast cells participate in endothelial activation. Human mast cell tryptase has been shown to induce or potentiate IL-8 and ICAM-1 expression in bronchial epithelial cells [39], IL-8 production in endothelial cells [40], collagen synthesis and chemotaxis in fibroblasts [41], and to initiate angiogenesis [42]. Mast cell chymase and carboxypeptidase A degrade low-density lipoprotein [13, 14]. The mouse mast cell protease-6, which is homologous to human *β*-tryptase, induces infiltration of neutrophils *in vivo* and stimulates IL-8 production by endothelial cells [43]. Evidence also indicates the presence of increased mast cell numbers and extracellular tryptase within the atherosclerotic plaques during early stages of calcification [10]. Results presented in Figure 5(c) demonstrate that incubation of HCAEC with tryptase results in significant increase in the production of IL-6 by HCAEC, which was further enhanced by the presence of LPS in the cell culture. These results suggest that tryptase and LPS can act in concert to amplify inflammatory response in the endothelium.

Histamine is well recognized for its role in the regulation of vasodilatation [44, 45] and endothelial cell functions [26]. A direct relationship between histamine and vascular inflammation is evident from the increased levels of histamine in the coronary arteries of patients with ischemic heart disease [2] and by the elevated levels of histamine in the coronary circulation of patients with variant angina [24]. The ability of mast cell-derived histamine to induce IL-6 production by HCAEC suggests that it is an important inflammatory mediator in the coronary vasculature. The inflammatory and hypersensitivity responses elicited by histamine are mediated through a family of four distinct G-protein-coupled receptors, H1R, H2R, H3R, and H4R [46–49]. The H1 receptor subtype is highly expressed in endothelial cells and smooth muscle cells. In the present study, IL-6 production induced by mast cells or by their synergistic action with LPS was partially inhibited by the H1R antagonist, diphenhydramine, but not by the H2R antagonist, famotidine (Figures 4(a) and 4(b)). Furthermore, the direct and synergistic effect of exogenous histamine on IL-6 production was significantly abrogated by the H1R antagonist (Figure 5(a)) but not by H2R antagonist (Figure 5(b)). However, a complete abrogation of the stimulatory effect of intact mast cells on IL-6 production was noted only when both the protease activity and the H1R function were blocked (Figure 4(c)).

Although recognized as an early phase reactant, the role of histamine in the delayed effects of inflammation on the vasculature is not clear. As discussed above, the direct cellular effect of histamine and its ability to synergize LPS-induced IL-6 production underscores its role in amplifying the inflammatory responses associated with infection. Prolonged interaction of histamine and LPS with endothelial cells results in vascular dysfunction. Other pro-inflammatory molecules such as C-reactive protein [32], oxysterols and TNF-*α* play a role in calcification via calcifying vascular cells, a type of vascular smooth muscle cells [33–35]. Calcification involves mechanisms similar to osteogenic mineralization [29] and the bone morphogen BMP-2 is expressed in calcifying atherosclerotic plaques [28]. BMP-2 is a powerful osteogenic morphogen which belongs to the transforming growth factor-beta (TGF-*β*) superfamily. Activated endothelial cells are known to release osteo/chondrocytic molecules such as BMPs [50, 51]. BMP-2 is a key participant in calcified atherosclerotic plaques [36], and its expression is enhanced by TNF-*α* or mechanical stimuli [52]. We have shown that mice deficient in mast cells and histamine are osteoporotic and osteoclast deficient, and they exhibit bone abnormalities [53] indicating a role for mast cells in the regulation of bone formation and calcification. Furthermore, a recent study demonstrated communication between BMP-2 and mast cells during heterotopic ossification [54]. Our present study demonstrating increased expression of BMP-2 mRNA and protein in histamine-treated HCAEC supports the idea that mast cell-derived histamine may modulate early events in vascular calcification. In this context, it is noteworthy that histamine has been shown to cause soft tissue calcification [55]. To our knowledge, this is the first study which links the role of histamine in the regulation of BMP-2 and may explain the bone abnormalities in mast cell deficiency.

## 5. Conclusions

The present study demonstrates that human mast cell line HMC-1 5C6 is capable of inducing the production of IL-6 by interacting with quiescent and LPS-induced HCAEC. The stimulatory effect of HMC-1 5C6 on LPS-mediated activation was synergistic in nature and was mediated by histamine and proteases. Exogenously added histamine and tryptase mimicked the direct and potentiating effects of LPS on IL-6 production. Another novel finding of this study is that histamine is capable of inducing the expression of the osteogenic protein BMP-2. Collectively, our results suggest that mast cell-derived histamine and proteases play an important role in vascular inflammation and calcification in addition to their well-recognized participation in allergic diseases.

## Figures and Tables

**Table 1:** HMC-1 5C6 directly and synergistically with LPS enhance endothelial cell production of IL-6.

Treatment	No LPS	LPS
Control	25 ± 3	546 ± 19
5C6 (7,500)	82 ± 6	1548 ± 68
5C6 (15,000)	170 ± 9	2238 ± 59
5C6-sonicate (7,500 cell equivalent)	118 ± 14	1380 ± 45
5C6-sonicate (15,000 cell equivalent)	227 ± 13	1842 ± 62

Confluent human coronary artery endothelial cells (15,000 cells/well) were incubated with the indicated number of human mast cells or their lysate after sonication in the presence or absence of LPS (100 ng/mL). After 20 h of incubation the culture supernatants were assayed for IL-6. HMC-1 5C6 cultured in the absence of HCAEC did generate IL-6 in the presence or absence of LPS. Each value presented is the mean ± SEM of quadruplicate determinations.

## References

[1] Fernex M (1968). *The Mast Cell System. Its Relationship to Atherosclerosis, Fibrosis and Eosinophils*.

[2] Kalsner S, Richards R (1984). Coronary arteries of cardiac patients are hyperreactive and contain stores of amines: a mechanism for coronary spasm. *Science*.

[3] Forman MB, Oates JA, Robertson D (1985). Increased adventitial mast cells in a patient with coronary spasm. *New England Journal of Medicine*.

[4] Dvorak AM (1986). Mast-cell degranulation in human hearts. *New England Journal of Medicine*.

[5] Gaça MDA, Pickering JA, Arthur MJP, Benyon RC (1999). Human and rat hepatic stellate cells produce stem cell factor: a possible mechanism for mast cell recruitment in liver fibrosis. *Journal of Hepatology*.

[6] Chiappetta N, Gruber B (2006). The role of mast cells in osteoporosis. *Seminars in Arthritis and Rheumatism*.

[7] Mekori YA, Metcalfe DD (1999). Mast cell-T cell interactions. *Journal of Allergy and Clinical Immunology*.

[8] Atkinson JB, Harlan CW, Harlan GC, Virmani R (1994). The association of mast cells and atherosclerosis: a morphologic study of early atherosclerotic lesions in young people. *Human Pathology*.

[9] Jeziorska M, Mccollum C, Woolley DE (1997). Mast cell distribution, activation, and phenotype in atherosclerotic lesions of human carotid arteries. *Journal of Pathology*.

[10] Jeziorska M, Mccollum C, Woolley DE (1998). Calcification in atherosclerotic plaque of human carotid arteries: associations with mast cells and macrophages. *Journal of Pathology*.

[11] DeSchryver-Kecskemeti K, Williamson JR, Jakschik BA, Clouse RE, Alpers DH (1992). Mast cell granules within endothelial cells: a possible signal in the inflammatory process?. *Modern Pathology*.

[12] Marks RM, Roche WR, Czerniecki M (1986). Mast cell granules cause proliferation of human microvascular endothelial cells. *Laboratory Investigation*.

[13] Kokkonen JO, Vartiainen M, Kovanen PT (1986). Low density lipoprotein degradation by secretory granules of rat mast cells. Sequential degradation of apolipoprotein B by granule chymase and carboxypeptidase A. *Journal of Biological Chemistry*.

[14] Lindstedt KA (1993). Inhibition of macrophage-mediated low density lipoprotein oxidation by stimulated rat serosal mast cells. *Journal of Biological Chemistry*.

[15] Li Y, Stechschulte AC, Smith DD, Lindsley HB, Stechschulte DJ, Dileepan KN (1997). Mast cell granules potentiate endotoxin-induced interleukin-6 production by endothelial cells. *Journal of Leukocyte Biology*.

[16] Jehle AB, Li Y, Stechschulte AC, Stechschulte DJ, Dileepan KN (2000). Endotoxin and mast cell granule proteases synergistically activate human coronary artery endothelial cells to generate interleukin-6 and interleukin-8. *Journal of Interferon and Cytokine Research*.

[17] Chi L, Stehno-Bittel L, Smirnova I, Stechschulte DJ, Dileepan KN (2003). Signal transduction pathways in mast cell granule-mediated endothelial cell activation. *Mediators of Inflammation*.

[18] Talreja J, Kabir MH, Filla MB, Stechschulte DJ, Dileepan KN (2004). Histamine induces Toll-like receptor 2 and 4 expression in endothelial cells and enhances sensitivity to Gram-positive and Gram-negative bacterial cell wall components. *Immunology*.

[19] Galli SJ (1993). Seminars in medicine of the Beth Israel Hospital, Boston: new concepts about the mast cell. *New England Journal of Medicine*.

[20] Schwartz LB, Irani AMA, Roller K (1987). Quantitation of histamine, tryptase, and chymase in dispersed human T and TC mast cells. *Journal of Immunology*.

[21] Reynolds DS, Gurley DS, Stevens RL, Sugarbaker DJ, Austen KF, Serafin WE (1989). Cloning of cDNAs that encode human mast cell carboxypeptidase A, and comparison of the protein with mouse mast cell carboxypeptidase A and rat pancreatic carboxypeptidases. *Proceedings of the National Academy of Sciences of the United States of America*.

[22] Vanderslice P, Ballinger SM, Tam EK, Goldstein SM, Craik CS, Caughey GH (1990). Human mast cell tryptase: multiple cDNAs and genes reveal a multigene serine protease family. *Proceedings of the National Academy of Sciences of the United States of America*.

[23] Urata H, Kinoshita A, Misono KS, Bumpus FM, Husain A (1990). Identification of a highly specific chymase as the major angiotensin II-forming enzyme in the human heart. *Journal of Biological Chemistry*.

[24] Sakata Y, Komamura K, Hirayama A (1996). Elevation of the plasma histamine concentration in the coronary circulation in patients with variant angina. *American Journal of Cardiology*.

[25] Takagishi T, Sasaguri Y, Nakano R (1995). Expression of the histamine H1 receptor gene in relation to atherosclerosis. *American Journal of Pathology*.

[26] Li Y, Chi L, Stechschulte DJ, Dileepan KN (2001). Histamine-induced production of interleukin-6 and interleukin-8 by human coronary artery endothelial cells is enhanced by endotoxin and tumor necrosis factor-*α*. *Microvascular Research*.

[27] Tan X, Essengue S, Talreja J, Reese J, Stechschulte DJ, Dileepan KN (2007). Histamine directly and synergistically with lipopolysaccharide stimulates cyclooxygenase-2 expression and prostaglandin I2 and E2 production in human coronary artery endothelial cells. *Journal of Immunology*.

[28] Bostrom K, Watson KE, Horn S, Wortham C, Herman IM, Demer LL (1993). Bone morphogenetic protein expression in human atherosclerotic lesions. *Journal of Clinical Investigation*.

[29] Towler DA, Shao JS, Cheng SL, Pingsterhaus JM, Loewy AP (2006). Osteogenic regulation of vascular calcification. *Annals of the New York Academy of Sciences*.

[30] Raveendran VV, Tan X, Sweeney ME (2011). Lipopolysaccharide induces H1 receptor expression and enhances histamine responsiveness in human coronary artery endothelial cells. *Immunology*.

[31] Blair RJ, Meng H, Marchese MJ (1997). Human mast cells stimulate vascular tube formation. Tryptase is a novel, potent angiogenic factor. *Journal of Clinical Investigation*.

[32] Oh J, Wunsch R, Turzer M (2002). Advanced coronary and carotid arteriopathy in young adults with childhood-onset chronic renal failure. *Circulation*.

[33] Tintut Y, Patel J, Territo M, Saini T, Parhami F, Demer LL (2002). Monocyte/macrophage regulation of vascular calcification in vitro. *Circulation*.

[34] Tintut Y, Alfonso Z, Saini T (2003). Multilineage potential of cells from the artery wall. *Circulation*.

[35] Demer LL, Tintut Y (2005). Return to ectopia: stem cells in the artery wall. *Arteriosclerosis, Thrombosis, and Vascular Biology*.

[36] Shao JS, Al Aly Z, Lai CF (2007). Vascular Bmp-Msx2-Wnt signaling and oxidative stress in arterial calcification. *Annals of the New York Academy of Sciences*.

[37] Shah BA, Li Y, Stechshulte DJ, Dileepan KN (1995). Phagocytosis of mast cell granules results in decreased macrophage superoxide production. *Mediators of Inflammation*.

[38] Weber S, Babina M, Krüger-Krasagakes S, Grützkau A, Henz BM (1996). A subclone (5C6) of the human mast cell line HMC-1 represents a more differentiated phenotype than the original cell line. *Archives of Dermatological Research*.

[39] Cairns JA, Walls AF (1996). Mast cell tryptase is a mitogen for epithelial cells: stimulation of IL-8 production and intercellular adhesion molecule-1 expression. *Journal of Immunology*.

[40] Compton SJ, Cairns JA, Holgate ST, Walls AF (1998). The role of mast cell tryptase in regulating endothelial cell proliferation, cytokine release, and adhesion molecule expression: tryptase induces expression of mRNA for IL-1*β* and IL-8 and stimulates the selective release of IL-8 from human umbilical vein endothelial cells. *Journal of Immunology*.

[41] Gruber BL, Kew RR, Jelaska A (1997). Human mast cells activate fibroblasts: tryptase is a fibrogenic factor stimulating collagen messenger ribonucleic acid synthesis and fibroblast chemotaxis. *Journal of Immunology*.

[42] Blair RJ, Meng H, Marchese MJ (1997). Human mast cells stimulate vascular tube formation. Tryptase is a novel, potent angiogenic factor. *Journal of Clinical Investigation*.

[43] Huang C, Friend DS, Qiu WT (1998). Induction of a selective and persistent extravasation of neutrophils into the peritoneal cavity by tryptase mouse mast cell protease 6. *Journal of Immunology*.

[44] Barnes PJ (1991). Histamine receptors in the lung. *Agents and Actions*.

[45] Hill SJ (1992). Multiple histamine receptors: properties and functional characteristics. *Biochemical Society Transactions*.

[46] Clark RAF, Gallin JI, Kaplan AP (1975). The selective eosinophil chemotactic activity of histamine. *Journal of Experimental Medicine*.

[47] Beer DJ, Matloff SM, Rocklin RE (1984). The influence of histamine on immune and inflammatory responses. *Advances in Immunology*.

[48] Jeannin P, Delneste Y, Gosset P (1994). Histamine induces interleukin-8 secretion by endothelial cells. *Blood*.

[49] Hough LB (2001). Genomics meets histamine receptors: new subtypes, new receptors. *Molecular Pharmacology*.

[50] Sorescu GP, Sykes M, Weiss D (2003). Bone morphogenic protein 4 produced in endothelial cells by oscillatory shear stress stimulates an inflammatory response. *Journal of Biological Chemistry*.

[51] Shin V, Zebboudj AF, Boström K (2004). Endothelial cells modulate osteogenesis in calcifying vascular cells. *Journal of Vascular Research*.

[52] Csiszar A, Smith KE, Koller A, Kaley G, Edwards JG, Ungvari Z (2005). Regulation of bone morphogenetic protein-2 expression in endothelial cells: role of nuclear factor-*κ*B activation by tumor necrosis factor-*α*, H_2_O_2_, and high intravascular pressure. *Circulation*.

[53] Stechschulte DJ, Sharma R, Dileepan KN (1987). Effect of the *mi* allele on mast cells, basophils, natural killer cells, and osteoclasts in C57Bl/6J mice. *Journal of Cellular Physiology*.

[54] Salisbury E, Rodenberg E, Sonnet C (2011). Sensory nerve induced inflammation contributes to heterotopic ossification. *Journal of Cellular Biochemistry*.

[55] Seyle H, Tuchweber B (1965). Mast cell products and tissue calcification. *Experimental Physiology*.

